# Association between Sports Participation, Factor VIII Levels and Bleeding in Hemophilia A

**DOI:** 10.1055/a-1983-0594

**Published:** 2022-12-31

**Authors:** Laura H. Bukkems, Olav Versloot, Marjon H. Cnossen, Siv Jönsson, Mats O. Karlsson, Ron A.A. Mathôt, Kathelijn Fischer

**Affiliations:** 1 Hospital Pharmacy-Clinical Pharmacology, Amsterdam University Medical Center, Noord-Holland, The Netherlands; 2 Center for Benign Haematology, Thrombosis and Haemostasis, Van Creveldkliniek, University Medical Center Utrecht, University Utrecht, Utrecht, The Netherlands; 3 Department of Physiotherapy, Institute of Movement Studies, University of Applied Science, Utrecht, The Netherlands; 4 Department of Pediatric Hematology and Oncology, Erasmus University Medical Center - Sophia Children's Hospital Rotterdam, Rotterdam, The Netherlands; 5 Department of Pharmacy, Uppsala University, Uppsala, Sweden

**Keywords:** bleeding, hemophilia A, prophylaxis, repeated time-to-event, sports

## Abstract

**Background** Little is known on how sports participation affects bleeding risk in hemophilia. This study aimed to examine associations between sports participation, factor VIII (FVIII) levels and bleeding in persons with hemophilia A.

**Methods** In this observational, prospective, single-center study, persons with hemophilia A who regularly participated in sports were followed for 12 months. The associations of patient characteristics, FVIII levels, and type/frequency of sports participation with bleeding were analyzed by repeated time-to-event modelling.

**Results** One hundred and twelve persons (median age: 24 years [interquartile range:16–34], 49% severe, 49% on prophylaxis) were included. During follow-up, 70 bleeds of which 20 sports-induced were observed. FVIII levels were inversely correlated with the bleeding hazard; a 50% reduction of the baseline bleeding hazard was observed at FVIII levels of 3.1 and a 90% reduction at 28.0 IU/dL. The bleeding hazard did not correlate with sports participation. In addition, severe hemophilia, prestudy annual bleeding rate, and presence of arthropathy showed a positive association with the bleeding hazard.

**Conclusion** This analysis showed that FVIII levels were an important determinant of the bleeding hazard, but sports participation was not. This observation most likely reflects the presence of adequate FVIII levels during sports participation in our study. Persons with severe hemophilia A exhibited a higher bleeding hazard at a similar FVIII levels than nonsevere, suggesting that the time spent at lower FVIII levels impacts overall bleeding hazard. These data may be used to counsel persons with hemophilia regarding sports participation and the necessity of adequate prophylaxis.

## Graphical abstract

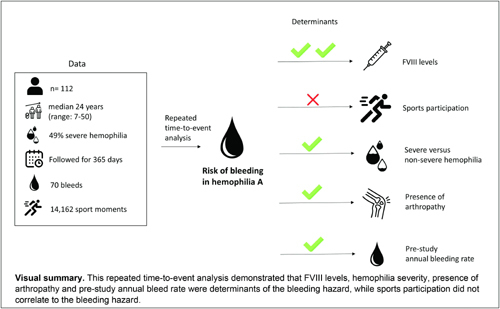

## Introduction

Persons with hemophilia A suffer from factor VIII (FVIII) deficiency and impaired hemostasis, resulting in spontaneous and/or trauma-related bleeding. Bleeding characteristically occurs in joints and muscles and eventually results in hemophilic arthropathy. Most severe and some moderate persons with hemophilia A are treated prophylactically with FVIII replacement therapy. For mild and most moderately affected persons, on-demand treatment is usually used. Recently, also nonreplacement therapies have been introduced. 1

Historically, only low-impact sports were recommended for persons with hemophilia due to a perceived increased bleeding risk when engaging in high-risk sports activities. 2 This advice has contributed to poor exercise performance and impaired muscle strength in persons with hemophilia as reported in early studies. 3 4 5 The widespread availability of factor concentrates in well-resourced countries and the introduction of prophylaxis have however enhanced the ability for sports participation for all persons with hemophilia. This is of importance as adequate physical activity overall reduces risk of chronic diseases and all-cause mortality. 6 7 Later studies conducted in settings in which adequate prophylaxis and on-demand treatment were available have demonstrated similar sports participation, physical fitness, and muscle strength in persons with hemophilia in comparison to the general population. 8 9 10 However, whether similar sports participation in persons with hemophilia as in the general population leads to a higher bleeding risk remains unanswered. Presumably, the risk of bleeding during sports participation is dependent on the achieved factor level.

Only a few studies have assessed the relationship between bleeding risk and physical activity. 11 12 Ross et al 11 observed no impact of athletic participation on joint outcomes. Tiktinsky et al 12 reported a higher bleeding rate during vigorous exercise in persons with severe hemophilia who were treated on demand. Only Broderick et al 13 examined the effect of factor levels and sports participation on bleeding risk and reported a moderate relative increase in bleeding risk during vigorous physical activities together with a reduction of bleeding at increasing FVIII levels. This study included only children with severe and moderate hemophilia, leaving a knowledge gap for both adult and mild hemophilia patients.

The association between sports participation, FVIII levels, and bleeding hazard can be modeled with a parametric repeated time-to-event (RTTE) model. This technique can characterize the occurrence of repetitive events (bleeding) over time and can be used to examine the association of various patient factors with the bleeding hazard. 14 15 16 The aim of this study was to examine the influence of sports participation and FVIII levels on the bleeding hazard in persons with hemophilia A in the current treatment setting in the Netherlands using an RTTE analysis.

## Methods

A detailed method description is presented in the Supplementary Material (available in the online version) .

### Data

In this observational, prospective, single-center study from University Medical Center Utrecht, persons with hemophilia who participated in sports at least once weekly were followed for 12 months (SPRAIN study). Participants were contacted bi-weekly to enquire about bleeds and injuries, including information on nature, mechanism, involvement of sports participation, and details of last factor concentrate administration. Data on prophylactic treatment regimen, body mass index (BMI), presence of arthropathy, and prestudy annual bleeding rate (ABR) were extracted from electronic patient files. Physical activity was assessed using a 1-week training diary and activity tracker. The sports described were assumed to be constant during the entire study period, with exception of summer and winter recess.

The study was registered in the Dutch Trial register under NTR6769 ( https://trialsearch.who.int/ ). The Medical Ethical Committee approved the study (IRB number: 18–141). Informed consent was obtained from all study participants and data were collected in accordance with the Declaration of Helsinki.

### Development of Repeated Time-to-Event Model

**Fig. 1:**
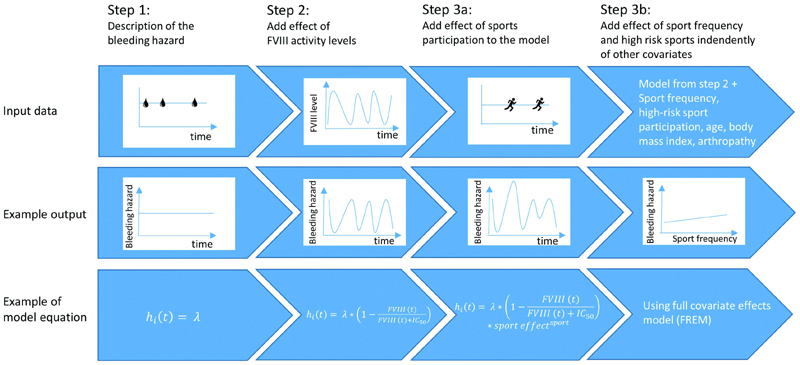
Visual description of the workflow to develop the repeated time-to-event (RTTE) model to evaluate the association between sports participation, FVIII levels, and bleeding. In the example equations, *h _i_* ( *t* ) describes the individual bleeding hazard at time *t* , λ the baseline bleeding hazard, FVIII, FVIII activity level at time *t* , IC _50_ the FVIII activity level at which 50% of the maximum inhibition on the bleeding hazard occurs, “sport effect” describes the change in bleeding hazard during sports, and “sport” is equal to 1 during a sports exposure and zero when not participating in sports. FVIII, factor VIII.

The modelling process is visualized in Fig. 1 . The probability of bleeding over time was analyzed using an RTTE model, which is a parametric survival method. 14 15 In the RTTE model, the bleeding probability over time is estimated by the parametric hazard function. Exponential, Gompertz, and Weibull hazard functions were evaluated to describe how the hazard varied over time. Inter-individual variability on the overall bleeding hazard was considered.

The effect of FVIII levels on the bleeding hazard was assessed by a maximum inhibition ( *I*_max_ ) model (Step 2 in Fig. 1 ). FVIII level was assumed to be constant for persons treated on demand, and was described by the lowest measured endogenous FVIII level. For persons treated prophylactically with data on FVIII levels available, individual pharmacokinetic (PK) parameters were estimated with the Web Accessible Population Pharmacokinetic Service (WAPPS) online tool using Bayesian forecasting. 17 18 19 For people lacking information on FVIII levels, individual PK parameters were estimated based on FVIII concentrate, age, bodyweight, and blood group using the population PK models applied by WAPPS. 17

The association between bleeding and sports participation was evaluated using two different methods. First, sports participation was incorporated as a time-varying covariate (set to 1 or 0 depending on exposure or not) in the data set, and the effect of sports participation on the bleeding hazard was evaluated (Step 3a in Fig. 1 ).

Second, a full random-effects model (FREM) was used (Step 3b in Fig. 1 ). The FREM characterizes the correlation between the bleeding hazard and all covariates of interest independently. 20 Examined covariates in the FREM were BMI, ABR, presence of arthropathy, endogenous FVIII level, sports frequency per month, and participation in high-risk sports. 20

### Model Development and Assessment

Model building was performed using nonlinear mixed-effect modelling in NONMEM v7.4.1. 22 Model evaluation was performed based on comparison of the observed and model-simulated Kaplan–Meier curves, scientific plausibility of the parameter estimates, their standard error, and the objective function value via the likelihood ratio test.

## Results

### Data

Patient and treatment characteristics are presented in Table 1 . One hundred and twelve persons with hemophilia A of which 13 children <12 years were included. Fifty-five had severe- (endogenous FVIII <1 IU/dL), 8 moderate (endogenous FVIII ≥1 and ≤5 IU/dL), and 49 had mild hemophilia (endogenous FVIII >5 IU/dL). In total, around half of the study population (49%) were treated prophylactically with FVIII concentrate, while the others used on-demand treatment. One person with moderate hemophilia was treated with prophylaxis and one person with severe hemophilia was treated on demand. FVIII levels were available for 23 (42%) persons on prophylaxis, for the other 32 persons on prophylaxis, FVIII levels were unavailable. Sports activities were performed a median of 13 times per month (range: 2–33) and 59% participated in high-risk sports. During the follow-up period, 167 injuries and 70 bleeds were reported, of which 35 (50%) were joint bleeds and 20 (29%) were sports-induced bleeds. Bleeds were mostly self-diagnosed by study participants, but 25 bleeds (36%) were evaluated by a medical professional.

**Table 1:** Patient and treatment characteristics

Characteristic	Number (percentage) or median [IQR] (range)
Patients	112
Age (y)	24.1 [16.0–33.7] (7.2–49.6)
Weight (kg)	77.0 [62.8–85.3] (24.0–135.0)
Body mass index (kg/m ^2^ )	22.5 [19.5–25.1] (14.2–38.5)
Hemophilia severity	
Severe (FVIII < 1 IU/dL)	55 (49%)
Moderate (FVIII ≥ 1 and ≤ 5 IU/dL)	8 (7%)
Mild (FVIII > 5 IU/dL)	49 (44%)
Endogenous FVIII level nonsevere (IU/dL)	15 [10–17] (2–29.0)
Sports frequency (per month)	13 [9–17] (2–33)
Participation in high-risk sports	66 (59%)
Follow up (d)	365 [365–365] (365–365)
Hemophilia joint health score	0 [0–3] (0–44)
Pre-existing arthropathy	22 (20%)
Treatment specifications	
Prophylaxis	55 (98% of severe patients)
Median FVIII dose (IU/kg/wk)	43.1 [36.0–53.9] (11.7–89.5)
Factor concentrate	
Standard half-life FVIII a	40 (73%)
Extended half-life FVIII b	15 (27%)
Bleeding specifications	
Bleeds ( *n* observed)	70
Joint bleeds	35 (50%)
Sports-induced bleed	20 (29%)
ABR before study inclusion	0 [0–1] (0–9)
AJBR before study inclusion	0 [0–0] (0–4)
ABR during study	0 [0–1] (0–5)

Abbreviations: ABR, annual bleeding rate; AJBR, annual joint bleed rate; FVIII, factor VIII.

a Advate, Kogenate, and Novoeight.

b Elocta. ABR describes the number of bleeds observed within 365 days.

### Development of Repeated Time-to-Event Model

An exponential hazard function described the bleeding data best, indicating a constant bleeding hazard over time. For 18 bleeds (26%, all not sports-induced) the exact time of bleed was unknown, therefore, interval censoring over the day was applied. The effect of FVIII level on the bleeding hazard was statistically significant ( *p* < 0.001) and was described with an *I*_max_ model, showing a higher bleeding hazard at lower FVIII levels ( Supplementary Fig. S1 [available in the online version]). The final individual hazard function was described by Equation 1 :



in which *h _i_* ( *t* ) describes the individual bleeding hazard at time *t* , λ the bleeding hazard in the absence of FVIII, FVIII the FVIII level at time *t* , IC _50_ the FVIII level at which 50% of the maximum inhibition on the bleeding hazard occurs, and η is a random effect describing the inter-individual variability in bleeding hazard.

**Fig. 2:**
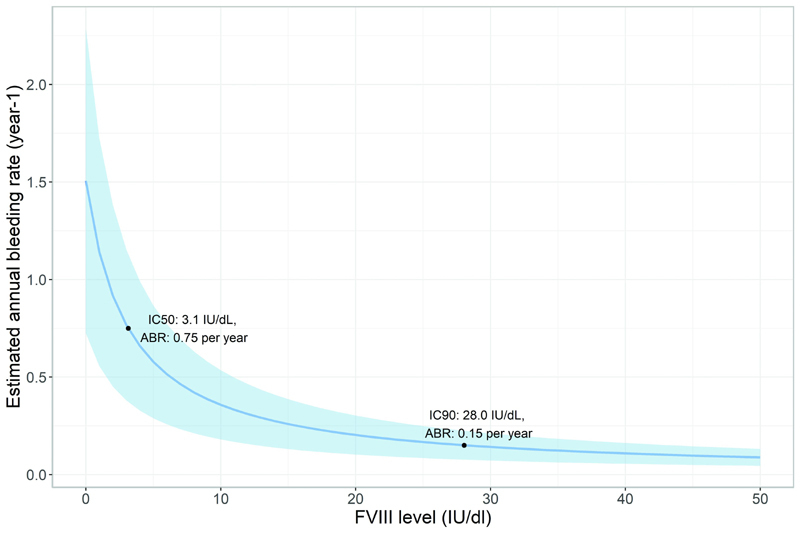
Relationship between factor VIII (FVIII) level and estimated annual bleeding rate (ABR). The *solid blue line* gives the median relation (based on the estimated model parameters) and the shaded area the 95% confidence interval (based on the relative standard errors of the parameter estimates). The IC50 and IC90 depict the FVIII level at which 50% or 90% of the maximal protective effect occur, respectively. A median patient in this dataset with a constant FVIII level of 3.1 IU/dL will experience 0.75 bleeds per year.

The parameter estimates of the model are presented in Table 2 . The estimated bleeding hazards can be interpreted as the estimated ABR when a person has a constant FVIII level. As a result, a median person with a constant FVIII level of 0, 1, 10, or 20 IU/dL will experience 1.5, 1.1, 0.4, or 0.2 bleeds per year, respectively. The estimated bleeding hazards for other FVIII levels are visualized in Fig. 2 .

**Table 2:** Parameter estimates of the final repeated time-to-event (RTTE) model

Parameter	Estimate	95% CI
Bleeding hazard at FVIII 0 IU/dL (derived) (y ^-1^ )	1.50	–
Bleeding hazard at FVIII 1 IU/dL (y ^-1^ )	1.14	0.56–1.72
Bleeding hazard at FVIII 10 IU/dL (derived) (y ^-1^ )	0.36	–
Bleeding hazard at FVIII 20 IU/dL (y ^-1^ )	0.20	0.10–0.30
IC50 (derived) (IU/dL)	3.12	–
IC90 (derived) (IU/dL)	28.0	–
Inter-individual variability of bleeding hazard (CV%)	92.4	48.9–135.9

Abbreviations: CI, confidence interval; CV, coefficient of variation calculated as  , shrinkage of inter-individual variability of bleeding hazard was 50%; IC50, FVIII activity level resulting in 50% reduction of the baseline bleeding hazard at a FVIII level of 0 IU/dL; IC90, FVIII activity level resulting in 90% reduction of the baseline bleeding hazard at a FVIII level of 0 IU/dL. The estimated bleeding hazards at 0, 1, 20, and 20 IU/dL can be interpreted as the median estimated annual bleeding rate when a patient has the respective constant FVIII level. A FVIII level of 3.12 IU/dL was found to reduce the median baseline annual bleeding rate of 1.50 per year to 0.75 per year.

Compared to persons with severe hemophilia and no measurable FVIII level, the ABR was reduced by 50% at a FVIII level of 3.1 IU/dL, by 75% at a FVIII level of 9.3 IU/dL, and by 90% at a FVIII level of 28.0 IU/dL. The inter-individual variability of bleeding hazard was high (coefficient of variation of 92.4%), demonstrating that people with similar FVIII levels presented with a varying number of bleeds. For instance, ABR for persons with a constant FVIII level of 1 IU/dL is median 1.1 per year but the 95% prediction interval was 0.2 to 5.3 per year.

In Supplementary Fig. S2 (available in the online version) , the observed Kaplan–Meier curves of the first, second, and third bleeds combined with 2.5th and 97.5th percentiles of the model-simulated Kaplan–Meier curves are presented. The simulated shaded areas cover the observed Kaplan–Meier curves, demonstrating that the model describes the bleeding probability observed in our data adequately. As described in the Methods section, we used two different strategies to estimate the individual PK parameters since FVIII levels were not available for every person. To analyze if these different strategies affected the estimates of the RTTE model, we developed RTTE models including only the prophylaxis patients with FVIII levels or only the prophylaxis patients without FVIII levels available. The results showed similar parameter estimates for patients on prophylaxis, indicating that these different methods did not affect the results ( Supplementary Table S1 [available in the online version] ).

### Sports Participation

During the study, 20 sports-induced bleeds occurred during 14,162 sport exposures. On average, subjects presented with a 21% higher FVIII level during sports than their average FVIII levels. The median estimated FVIII level during sports-induced bleeds was 5.9 IU/dL (range: 0–20 IU/dL), while these were 11.0 IU/dL (range: 0–95 IU/dL) during sports activities without occurrence of bleeding. When the median FVIII levels between sports-induced bleeds and during sport activities were compared for severe, moderate, and mild hemophilia patients separately, the difference was larger for persons with severe hemophilia (4.5 IU/dL during sports-induced bleeds and 8.6 IU/dL during sports activities without bleeding) than for mild hemophilia (14.0 IU/dL during sports-induced bleeds and 15.0 IU/dL during sports activities without bleeding).

In the first covariate analysis, sports participation was related to the bleeding hazard. Results showed that during sports participation the bleeding hazard did not change significantly, as inclusion of this covariate did not improve the goodness of fit ( *p* > 0.05).

**Fig. 3:**
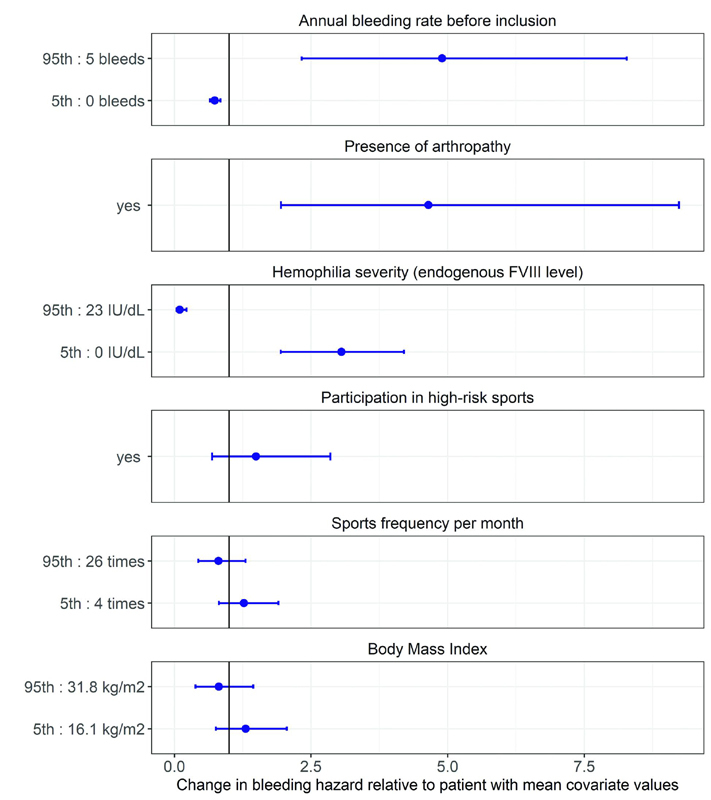
Effect of the examined patient characteristics (covariates) on the bleeding hazard. The change in bleeding hazard ( *dots* , point estimate) relative to a patient with mean covariate values is described for the 5th and 95th percentiles of the distribution of the examined covariates. The error bars present the uncertainty around the 5th and 95th percentile point estimates, given by the 90% confidence interval. The *solid line* at 1.0 indicates no change in the bleeding hazard relative to a patient with mean covariate values. The mean study patient presented with an annual bleeding rate of 0.8 per year, experienced no arthropathy, had an endogenous FVIII level of 7.1 IU/dL, did not participate in high-risk sports, played sports 13.6 times per month, and had a body mass index of 22.9 kg/m ^2^ . Covariates are ranked from covariates with the strongest correlation with bleeding hazard on top (based on point estimates) to no correlation with the bleeding hazard on the bottom.

The results of the second covariate analysis using the FREM methodology, estimated weak, statistically non-significant correlations between bleeding hazard and both (1) sports frequency per month and (2) participation in high-risk sports, as illustrated in Fig. 3 . In this figure, the 90% confidence interval whiskers cross the solid reference line of a mean participant, indicating that when sports frequency per month and participation in high-risk sports differ from this mean, there is no strong association with the bleeding hazard. Covariates that showed strong correlations with bleeding hazard and indicated an increased bleeding hazard were high ABR, presence of arthropathy, and severe hemophilia. For instance, pre-existing arthropathy resulted in a median 4.6 times higher bleeding hazard when compared to persons without pre-existing arthropathy. Consequently, persons with pre-existing arthropathy required a 4.6 times higher FVIII level to achieve a similar ABR to persons without pre-existing arthropathy.

## Discussion

This study is the first to evaluate the association of sports participation and FVIII levels with bleeding hazard in both severe and nonsevere hemophilia A in a wide age range. Bleeding hazard was predominantly determined by FVIII levels. A FVIII level of 3.1 IU/dL was found to reduce the ABR in the absence of FVIII by 50%, while a FVIII level of 28.0 IU/dL reduced this baseline ABR by 90%. No association between sports participation and bleeding hazard was observed in our study population, as neither frequency nor intensity of sports participation (low vs. high risk) showed an independent association with bleeding hazard. Other covariates independently associated with the bleeding hazard were ABR before study inclusion, presence of arthropathy, and hemophilia severity.

### Possible Explanations for Observed Results

How can we explain the lack of association between sports participation and bleeding hazard? We presume two main reasons may play a role. First, prophylactic treatment is personalized according to an individual's sports schedule and other physical activities, consciously targeting higher FVIII levels during sports. The median estimated FVIII levels during sports-induced bleeds were lower than the median FVIII levels observed during sports activities in which no bleeding occurred (5.9 vs. 11.0 IU/dL), which suggests that higher FVIII levels protected against bleeding during sports activities. Second, as our study population regularly participated in sports, increased muscle mass and strength may also have protected against sports-induced bleeds, as physical fitness and muscle strength resulting from regular sports participation may help prevent bleeding. 23 24

In the covariate analysis, additional covariates showed independent associations with the bleeding hazard. As expected, a higher ABR before study inclusion and presence of arthropathy predicted a higher bleeding hazard. Hemophilia severity and thus endogenous FVIII levels were negatively associated with bleeding hazard, indicating a higher bleeding hazard for persons with lower endogenous FVIII levels. This suggests that persons with severe hemophilia have a higher bleeding hazard than persons with nonsevere hemophilia when similar FVIII levels are achieved. For example, when a person with severe hemophilia reaches a FVIII level of 20 IU/dL with the use of prophylaxis, his bleeding hazard will be higher at this time point than when a person with mild hemophilia has a FVIII level of 20 IU/dL. This finding seems to contradict the general view that prophylaxis is able to convert severe hemophilia into moderate hemophilia. 25 However, this study observation may be explained by the fact that persons with nonsevere hemophilia solely treated on demand in majority have stable endogenous FVIII levels. Contrastingly, persons with severe hemophilia on prophylaxis experience fluctuating FVIII levels, often returning to FVIII levels under or around 1 IU/dL, not seen in persons with nonsevere hemophilia A. These repetitive low FVIII trough levels, seen before administration of prophylaxis, are expected to increase the overall bleeding hazard. This observation is in accordance with the observation of Collins et al, which observed that increased time periods spent with FVIII levels <1 IU/dL were associated with an overall higher bleeding risk, and emphasizes the importance of FVIII trough levels and/or time spent under a certain FVIII level during prophylactic treatment. 26

### Study Strengths and Limitations

Strengths of this study include the bi-weekly contact with participants to gather information on bleeding and injuries, minimizing recall bias. Furthermore, for this analysis, RTTE modelling was used, which is a powerful method to describe time-varying events such as bleeding over time and its association with FVIII levels.

Importantly, we underline that study results cannot be directly extrapolated to all persons with hemophilia A, as this study only included persons who regularly participated in sports. This may have introduced selection bias, as persons experiencing many and severe bleeds due to sports participation may have ended sports activities and could therefore not be included in this study. On the other hand, it has been established that the majority (± 70%) of Dutch adults and Dutch children with hemophilia play sports. 10 Furthermore, in settings in which FVIII prophylaxis regimens are not consciously adjusted to sports schedules, an association between sports participation, factor levels, and bleeding may be easier identified. Our findings may also be limited due to incomplete data on exact FVIII timing and doses, as well as sports activities during the follow-up period as the training diary was only completed for a 1-week period due to practical considerations, which may be too short and less representative. Importantly, exact details of the last FVIII dose and details of sports participation were recorded for each bleed. During other periods, standard FVIII dosing and sports regimens were presumed. Lastly, bleeds were generally self-reported by study participants and only 36% of the bleeds were evaluated by a medical specialist.

### Comparison to Other Studies

In our study, the bleeding hazard for a constant FVIII level of 0.5 IU/dL was estimated to give 1.3 bleeds per year (95% CI: 0.5–2.1), which is lower than the 2.8 bleeds per year with a constant FVII level of 0.5 IU/dL estimated by Abrantes et al 16 in an RTTE analysis of the BAY 81-8973 clinical trial data in severe hemophilia. Concomitantly, the IC50 estimate in this current study of 3.1 IU/dL was also lower than the IC50 value of 10.2 IU/dL reported by Abrantes et al. These differences may be due to the lower overall number of bleeds observed in our study population, caused by differences in intensity of treatment, different evaluations of bleeding events as well as the inclusion of persons with nonsevere hemophilia. Importantly, the study of Abrantes et al 16 did not include sports participation as a covariate in the analysis.

Broderick et al 13 examined sports participation and bleeding in 104 boys with severe hemophilia and observed a moderate relative increase in the bleeding risk immediately following vigorous physical activities, while we were not able to identify an association between participation in high-risk sports and bleeding hazard. Possibly, this is caused by the differences in statistical power (436 vs. 70 bleeds), population (children vs. all ages), different statistical methods, and/or a different FVIII treatment regimen.

### Conclusion

We conclude that in this study, FVIII levels are an important determinant for the bleeding hazard, while sports frequency, participation in high-risk sports, and sports participation were not associated with the bleeding hazard. The low number of sports-induced bleeds complicated analyses of the association between FVIII levels and bleeding during sports participation. However, based on the association between FVIII levels and bleeding during the entire study, it could be derived that FVIII levels above 28.0 IU/dL decrease the ABR by at least 90% compared to when no FVIII level is measurable. Furthermore, a higher bleeding hazard was observed for persons with a high ABR or persons with pre-existing arthropathy, suggesting the need for higher FVIII levels. Moreover, in persons with severe hemophilia, a higher bleeding hazard was observed than in nonsevere hemophilia at similar FVIII levels. Importantly, this finding suggests that lower FVIII levels in between prophylactic infusions impact the overall bleeding hazard. These data provide important information for counselling regarding sports participation and underline the need for adequate prophylaxis as well as adequate targets for replacement and nonreplacement therapy.
