# Supplementary material for: Association between Sports Participation, Factor VIII Levels and Bleeding in Hemophilia A
Source: Thromb Haemost. 2022 Dec 31;123(3):317–25. doi: 10.1055/a-1983-0594 (PMC9981275; doi:10.1055/a-1983-0594)
Supplement: Supplementary file 1 — Supplementary Material [file 10-1055-a-1983-0594-s22070333.pdf]

Supplementary Methods

Data

In this observational, prospective, single-center Sports Participation and Injuries in people with hemophilia (SPRAIN) study from University Medical Center Utrecht (The Netherlands), persons with hemophilia who regularly participated in sports were followed for 1 year. For the present analysis, data from 13 people with hemophilia B were excluded, as the exposure–effect relation of factor VIII (FVIII) and factor IX concentrates could be different. Injuries and bleeds were assessed proactively, i.e., participants were contacted bi-weekly, and information about nature, involvement of sports participation, mechanism of injury leading to a bleed, and, in case of prophylactic treatment, last factor concentrate dose and timing of dosing and event were recorded. Bleeding was defined according to the International Society on Thrombosis and Haemostasis definitions.[1] A bleed was classified as occurring during sports when the bleed developed following a sports injury and required treatment with factor concentrates with or without a consultation with the hemophilia treatment center. Participation in high-risk sports was based on a National Hemophilia Foundation score >2.[2] The hemophilia joint health score was assessed at study initiation.[3,4]

Repeated Time-to-Event Model

A repeated time-to-event (RTTE) model is able to characterize the occurrence of time-varying events (bleeding over time) together with event predictors (e.g., factor activity levels and sports activities). In an RTTE model, the bleeding probability over time is described by the hazard function. First, a median bleeding hazard was estimated which describes the bleeding hazard for a median person using data from the whole population simultaneously. Differences in bleeding hazard between persons were evaluated by inclusion of the inter-individual variability on the hazard.

Exponential (Equation 1), Gompertz (Equation 2), and Weibull (Equation 3) hazard functions were tested to describe the distribution of time to bleeding. An exponential hazard function describes a constant hazard over time, while Gompertz and Weibull hazard functions can describe increasing or decreasing bleeding hazards over time.[5] The final individual hazard function was described by Equation 4:

$$h_i(t) = \lambda\left(1 - \frac{\mathrm{FVIII}\ (t)}{\mathrm{FVIII}\ (t) + \mathrm{IC}_{50}}\right) e^{\eta i} \ (1)$$

$$h(t) = \lambda \ (1)$$

$$h(t) = \lambda\, e^{\gamma\, t} \ (2)$$

$$h(t) = \lambda\gamma(\lambda t)^{\gamma-1} \ (3)$$

in which the bleeding hazard of the ith patient at time t is described by $h_i(t)$. λ describes the scale, γ the shape, FVIII the FVIII activity level at time t, IC_{50} the FVIII activity level at which 50% of the maximal inhibition on the bleeding hazard occurs and ηi the inter-individual variability in bleeding hazard with mean 0 and variance ω^2.

For persons not receiving a FVIII dose on the day of study initiation, FVIII levels at start of study were calculated based on the previous dose administered prior to study inclusion.

The λ and IC50 values were parameterized to describe the bleeding hazard for a FVIII level of 0.5 and 20 IU/dL following Equations 5 and 6.[6]

$$h_i(t) = \lambda\left(1 - \frac{\mathrm{FVIII}\ (t)}{\mathrm{FVIII}\ (t) + \mathrm{IC}_{50}}\right) e^{\eta i} \ (4)$$

$$\lambda = \frac{\lambda_{0.5}\ \lambda_{20}\ (0.5 - 20)}{(\lambda_{0.5}\ 0.5 - \lambda_{20}\ 20)} \ (5)$$

The survival function describes the probability of not having a bleed within a specific time interval. By taking the integral of the hazard, the cumulative hazard can be calculated, which is used to calculate the survival function (Equation 7):

$$IC50 = \frac{(\lambda_{0.5}\ 0.5 - \lambda_{20}\ 20)}{(\lambda_{0.5} - \lambda_{20})} \ (6)$$

in which the survival function of the ith patient within the time interval 0 to t_j is described by $S_i(t)$. In this example, 0 is taken as start of the time interval and t_j as the end of the time interval, and $h_i(t)$ is the individual bleeding hazard.

For some bleeds only the day of the bleeding was known, but not the exact time of the bleeding event. Interval censoring was applied for these bleeds. The probability that these bleeds occurred can be described by the probability that the event occurred between t_j and t_j +24 hours, following equation 8:

$$S_i(t) = e^{-\int_0^{t_j} h_i(t)\, dt} \ (7)$$

Covariate Analysis

A full random-effects model (FREM) was used to identify covariates with an effect on individual bleeding hazard.[7] This covariate analysis method can characterize the correlation between model parameters—such as the bleeding hazard—and all patient characteristics of interest simultaneously. Herewith, the correlation between the bleeding hazard (including the effect of FVIII levels) and sports activities can be evaluated independently of other patient factors.

Furthermore, problems with correlations between covariates and multiplicity are avoided with this method. Covariates are described by the mean and variance, handled as observations into the dataset. The mean is included as fixed effect and the variance as a random effect. The FREM model estimates the random effects of the parameters and covariates and the covariance between those two in a full covariance matrix. The covariance between the parameter and covariates describes the covariate effect. An exponential covariate parameter relationship was used.

During model development, we did not evaluate injuries as a covariate, as all bleeds except for four spontaneous bleeds were related to an injury. Furthermore, when an

 © 2022. The Author(s).

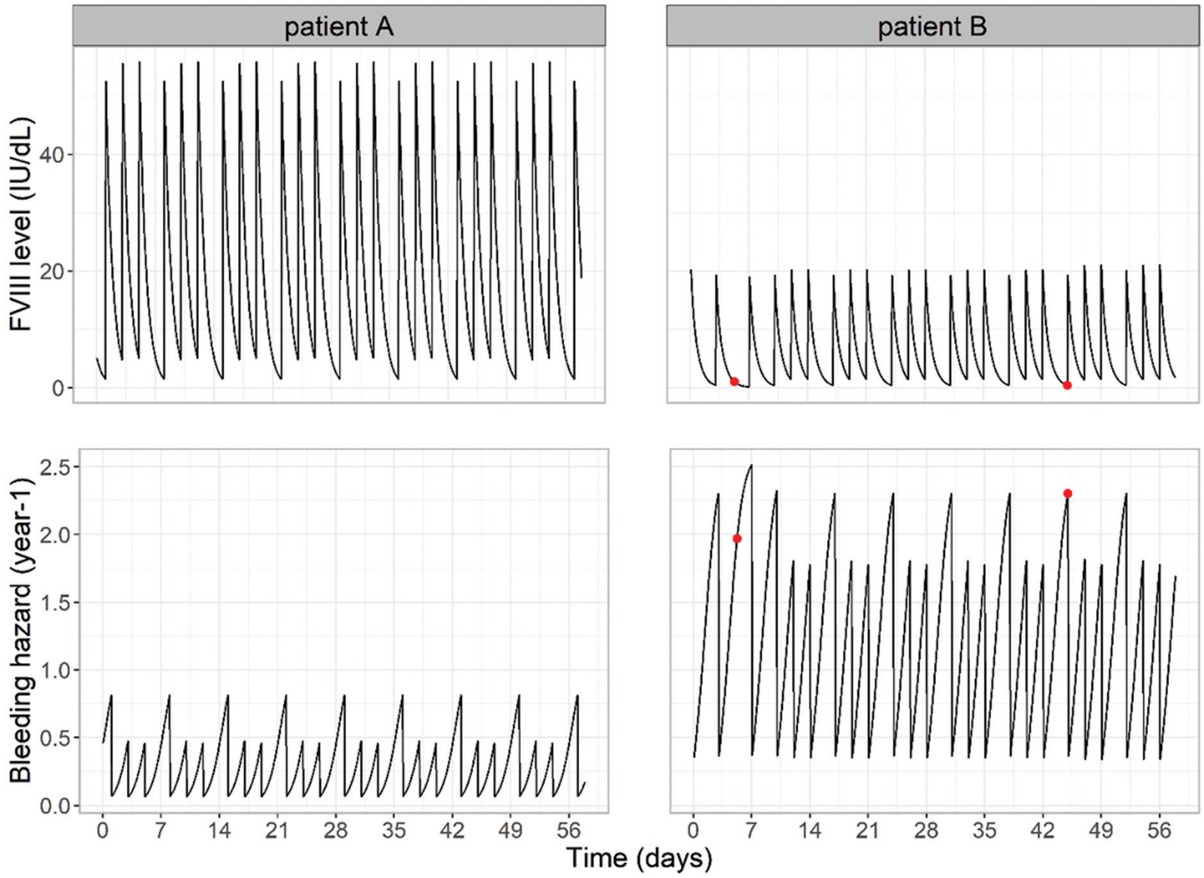

Supplementary Fig. S1 Illustration of the relationship between factor VIII (FVIII) levels and bleeding hazard for two individuals from the dataset. In the top panels, individual FVIII level over time is plotted, while the bottom panels show the corresponding model-predicted individual bleeding hazard. Patient A (10 years, 33 kg, treated with 3× per week 750 IU Elocta) did not experience any bleeds, while patient B (34 years, 73 kg, treated with 3× per week 1,000 IU Novoeight) experienced two bleeds (dots). The bleeding hazard is inversely related to the FVIII levels and is in general higher for patients who experience more bleeds.

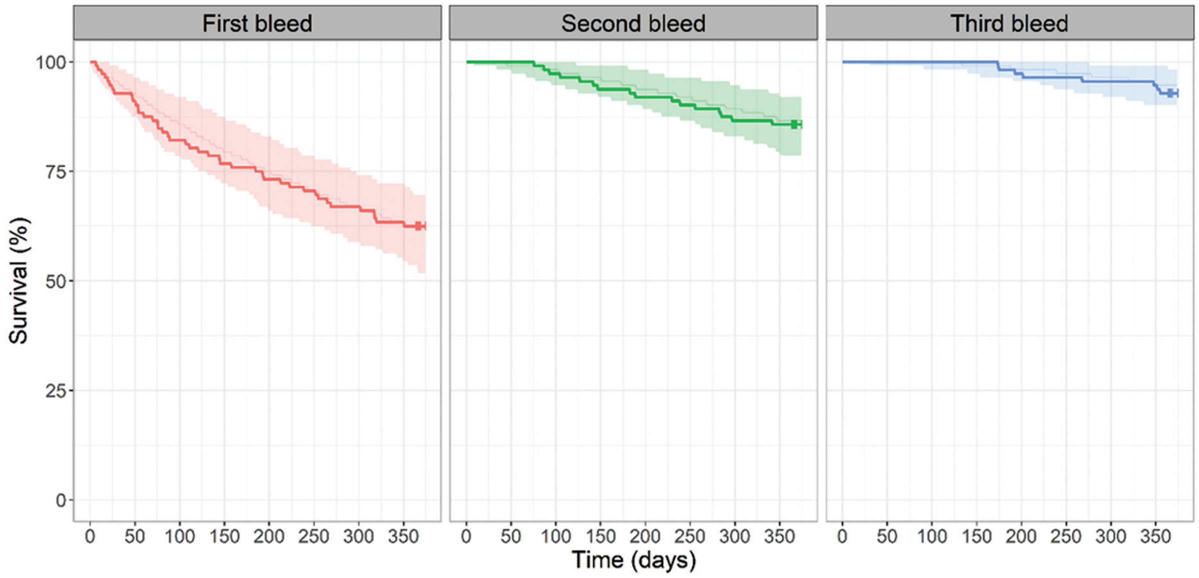

Supplementary Fig. S2 Kaplan–Meier curves of the first, second, and third bleeds (solid lines) combined with 2.5th and 97.5th percentiles of the model-predicted model predicted Kaplan–Meier curves (shaded area, *n* = 500 simulations). The shaded areas cover the Kaplan–Meier curves of observed bleeds, demonstrating that the developed model describes the bleeding probability in our data adequately.

 © 2022. The Author(s).

Supplementary Table S1 Parameter estimates and their 95% CI of the RTTE model in different patient groups

	All patients ($n=112$)	Prophylaxis patients with available FVIII levels ($n=23$)	Prophylaxis patients with no available FVIII levels ($n=32$)
Bleeding hazard at FVIII 1 IU/dL (y^{-1})	1.14 (0.56–1.72)	1.49 (0.18–2.8)	1.23 (0.42–2.03)
Bleeding hazard at FVIII 20 IU/dL (y^{-1})	0.20 (0.10–0.30)	0.25 (−0.01 to 0.51)	0.43 (0.02–0.84)
Inter-individual variability of bleeding hazard (CV%)	92.4 (48.9–135.9)	89.0 (−1.7 to 179.7)	79.0 (15.5–142.5)

Abbreviations: CI, confidence interval; RTTE, repeated time-to-event.

injury occurred without a bleed, the timing of the last concentrate dose was not explicitly recorded.

Model Development and Assessment

The RTTE model was developed in NONMEM (v7.4.1, Icon Development Solutions, Gaithersburg, Maryland, United States). The model was estimated with the Monte Carlo importance sampling assisted by mode a posteriori (IMPMAP) method. R v4.1.1, Piranha v2.9.9 and PsN v5.2.6 were used for data handling, visualization, model management, and evaluation.

References

1 Blanchette VS, Key NS, Ljung LR, Manco-Johnson MJ, van den Berg HM, Srivastava ASubcommittee on Factor VIII, Factor IX and Rare Coagulation Disorders of the Scientific and Standardization Committee of the International Society on Thrombosis and Hemostasis. Definitions in hemophilia: communication from the SSC of the ISTH. J Thromb Haemost 2014;12(11):1935–1939

2 Anderson A, Forsyth A. Playing It Safe: Bleeding Disorders, Sports and Exercise. New York, NY: National Hemophilia Foundation; 2017:1–72

3 Feldman BM, Funk S, Lundin B, Doria AS, Ljung R, Blanchette VInternational Prophylaxis Study Group (IPSG) Musculoskeletal measurement tools from the International Prophylaxis Study Group (IPSG). Haemophilia 2008;14(Suppl 3):162–169

4 Feldman BM, Funk SM, Bergstrom BM, et al. Validation of a new pediatric joint scoring system from the International Hemophilia Prophylaxis Study Group: validity of the hemophilia joint health score. Arthritis Care Res (Hoboken) 2011;63(02):223–230

5 Holford N. A time to event tutorial for pharmacometricians. CPT Pharmacometrics Syst Pharmacol 2013;2(05):e43

6 Abrantes JA, Solms A, Garmann D, Nielsen EI, Jönsson S, Karlsson MO. Relationship between factor VIII activity, bleeds and individual characteristics in severe hemophilia A patients. Haematologica 2020;105(05):1443–1453

7 Yngman G, Bjugård Nyberg H, Nyberg J, Jonsson EN, Karlsson MO. An introduction to the full random effects model. CPT Pharmacometrics Syst Pharmacol 2022;11(02):149–160

 © 2022. The Author(s).
